# *Pneumocystis* Pneumonia: Immunity, Vaccines, and Treatments

**DOI:** 10.3390/pathogens10020236

**Published:** 2021-02-19

**Authors:** Aaron D. Gingerich, Karen A. Norris, Jarrod J. Mousa

**Affiliations:** 1 Center for Vaccines and Immunology, College of Veterinary Medicine, University of Georgia, Athens, GA 30602, USA; arn80509@uga.edu (A.D.G.); kanorris@uga.edu (K.A.N.); 2 Department of Infectious Diseases, College of Veterinary Medicine, University of Georgia, Athens, GA 30602, USA

**Keywords:** *Pneumocystis jirovecii*, *Pneumocystis* pneumonia, fungal vaccines

## Abstract

For individuals who are immunocompromised, the opportunistic fungal pathogen *Pneumocystis jirovecii* is capable of causing life-threatening pneumonia as the causative agent of *Pneumocystis* pneumonia (PCP). PCP remains an acquired immunodeficiency disease (AIDS)-defining illness in the era of antiretroviral therapy. In addition, a rise in non-human immunodeficiency virus (HIV)-associated PCP has been observed due to increased usage of immunosuppressive and immunomodulating therapies. With the persistence of HIV-related PCP cases and associated morbidity and mortality, as well as difficult to diagnose non-HIV-related PCP cases, an improvement over current treatment and prevention standards is warranted. Current therapeutic strategies have primarily focused on the administration of trimethoprim-sulfamethoxazole, which is effective at disease prevention. However, current treatments are inadequate for treatment of PCP and prevention of PCP-related death, as evidenced by consistently high mortality rates for those hospitalized with PCP. There are no vaccines in clinical trials for the prevention of PCP, and significant obstacles exist that have slowed development, including host range specificity, and the inability to culture *Pneumocystis* spp. in vitro. In this review, we overview the immune response to *Pneumocystis* spp., and discuss current progress on novel vaccines and therapies currently in the preclinical and clinical pipeline.

## 1. Introduction

*Pneumocystis* pneumonia was first documented in the early 1900s, yet was first identified in humans following the conclusion of World War II in malnourished infants who had severe pneumonia [1]. *Pneumocystis* spp. were initially classified as protozoan parasites; however, it was determined in 1988 via RNA sequencing that *Pneumocystis* is an ascomycetous fungus [2]. Since 1988, five different species have been fully identified, including *Pneumocystis carinii* and *Pneumocystis wakefieldiae* in rats, *Pneumocystis murina* in mice, *Pneumocystis oryctolagi* in rabbits, and *Pneumocystis jirovecii* (formerly *carinii*) in humans [3]. *P. jirovecii* is the etiological agent of *Pneumocystis* pneumonia (PCP), a life-threatening pneumonia occurring primarily in immunocompromised individuals. PCP accounts for an estimated 10,000 hospitalizations [4] in the United States and >400,000 cases worldwide each year [5]. A sharp increase in cases of PCP was observed during the early years of the human immunodeficiency virus (HIV)/acquired immunodeficiency disease (AIDS) epidemic, as PCP was the most common defining opportunistic infection of individuals with HIV [6]. As antiretroviral therapy became more effective and widespread, the incidence of PCP decreased substantially; however, PCP remains a significant cause of mortality and morbidity for those with HIV [7]. The majority of new HIV infections, and thus HIV-related PCP cases, occur in developing countries, and an increase in the rate of PCP was observed from 2002 to 2010 [8].

PCP also has increased incidence in cancer patients undergoing immunosuppressive regimens [9], organ transplant recipients [10], and those on immunomodulating drugs [11,12,13,14]. Currently, percentages of PCP cases in HIV+ vs. HIV− populations are approximately 40% and 60%, respectively [14]. HIV-negative patients who develop PCP are at higher risk of death than those who are HIV+, with a mortality rate above 30% [15]. PCP can also progress more rapidly in HIV− patients and remains difficult to diagnose. HIV− patients typically have a more functional immune system than those who are HIV+, and thus, more severe PCP-associated inflammation [16]. In addition, PCP is correlated with the development and severity of chronic obstructive pulmonary disease (COPD) in patients with and without HIV infection [17,18,19,20,21,22,23,24].

One of the most challenging aspects in treating PCP is the presence of cholesterol in the cell membrane, which cannot be targeted by common antifungals such as amphotericin B and azoles [25]. Based on the high mortality rate with both HIV and non-HIV associated PCP [11,26], the need for novel therapeutic options and an effective vaccine remains. Currently, several drugs, vaccines, and even antibody therapies are being pursued. The host range specificity of *Pneumocystis* spp. has made it difficult to translate research in animal models into humans, and the inability to culture the organism in a lab setting has further impeded progress. In this review, we will summarize the immune response to PCP in the context of promising prevention and treatment options.

## 2. Development of PCP and the Immune Response

*Pneumocystis* spp. have a penchant to infect the lungs of immunocompromised individuals. Development of PCP has been hypothesized to be a reactivation event of a latent infection within a host when the immune system becomes compromised [27]. However, current thought is that person-to-person transmission is the likely culprit of new infections [28,29,30,31,32]. Based on microscopic examinations, *Pneumocystis* organisms attach to type I alveolar epithelium, which allows the fungus to transition from its small trophic form to the larger cystic form [33]. *Pneumocystis* is primarily an alveolar pathogen, but in very rare cases of severely immunocompromised individuals, it can disseminate from the lungs to other regions, including the central nervous system, bone marrow, lymph nodes, eyes, gastrointestinal tract, thyroid, liver, spleen, and kidney [34,35,36,37]. Adherence of *Pneumocystis* to alveoli is not the singular cause of diffuse alveolar damage, but rather, it is the host inflammatory response that can cause significant lung injury and impaired gas exchange, potentially leading to hypoxia and respiratory failure [38]. In the section below, we briefly summarize several aspects of the immune response to *Pneumocystis* infection based on immune cell type.

### 2.1. CD4+ T-Cells

CD4+ T-cells play a key role in the immune response to PCP by leading the recruitment of macrophages and monocytes to the lungs. In patients with HIV/AIDS, lower CD4+ T cell levels are correlated with a higher incidence of developing PCP [39]. Furthermore, depletion of CD4+ T cells in mice leads to the development of PCP [40], and in SCID mice, which lack a T and B lymphocyte response, spontaneous *Pneumocystis* infection is observed within three weeks [41]. SCID mice are able to salvage their ability to clear *Pneumocystis* infection effectively when reconstituted with CD4+ T cells from immunocompetent mice [42]. In addition, several studies have shown that proper CD4+ T-cell signaling is necessary for control of *Pneumocystis* infection. For example, mice deficient in both CD2 and CD28 (CD2/CD8) exhibit susceptibility to spontaneous *P. carinii* infection. In contrast, mice deficient in only CD2 or CD28 are not susceptible to spontaneous infection [43], although CD28-deficient mice can be infected when directly inoculated. However, CD28-deficient and CD2/CD28 deficient mice are able to clear infection, although such clearance is delayed. CD28-deficient mice have increased IFN-γ [43], which is important for limiting *Pneumocystis*-associated inflammation, but not in the resolution of infection [44,45,46,47]. In contrast, TNF-α is critical for clearance of PCP [44]. Supporting these observations, CD2/CD28-deficiency leads to reduced anti-*Pneumocystis* antibody titers. In addition, CD28-deficient mice have an increase in IL-10 levels [43], which is notable since IL-10-deficient mice have improved clearance of *Pneumocystis* infection [48]. Furthermore, the absence of IL-10 is correlated to improved *Pneumocystis* clearance and an increase in the presence of CD4+ T cells, CD8+ T-cells, and neutrophils in the lungs [48].

### 2.2. CD8+ T Cells

The role of CD8+ T cells in the clearance of *Pneumocystis* infection remains uncertain as evidence both for and against the role of this cell type has been demonstrated. CD4+ T cell-depleted mice modified to upregulate IFN-γ have improved clearance of *Pneumocystis* organisms, as well as an increase in recruited CD8+ T cells and NK cells to the lung [49]. CD8+ T cells derived from *Pneumocystis*-infected mice and stimulated with IFN-γ improve organism killing by macrophages [50]. Furthermore, administration of human IL-7 to CD4+ T cell-depleted mice results in an increase in IFN-γ-positive CD8+ T cell recruitment to the lungs, which is correlated to *Pneumocystis* clearance [51]. In a study assessing secondary immune responses to *Pneumocystis*, mice depleted of CD8+ T cells between first and second *Pneumocystis* challenge have higher fungal burden compared to CD4+ T cell-depleted mice [52], as previous work has shown that CD4+ T cells are not required for memory recall responses against *Pneumocystis* [53]. In contrast to these studies supporting the involvement of CD8+ T cells in *Pneumocystis* clearance, no difference in the organism lung burden is observed when both CD8+ and CD4+ T cells are depleted compared to CD4+ T cell-depleted mice, and reconstitution with sensitized CD8+ T cells leads to enhanced pulmonary injury [54].

### 2.3. Macrophages

Macrophages play a key role in clearing *Pneumocystis* infection, as alveolar macrophage depletion leads to an increase in *Pneumocystis* burden in the lungs of rats [55]. Alveolar macrophages are the primary responders to the organism, and once stimulated by IFN-γ from CD4+ T-cells, they are responsible for phagocytosis and killing of *Pneumocystis* organisms. In addition, alternatively activated macrophages are potent effector cells that can lead to robust organism killing [56,57]. However, *Pneumocystis* can subvert the effectiveness of macrophages by causing them to undergo apoptosis [58], as one study demonstrated that infected animal bronchoalveolar lavage (BAL) samples have high levels of polyamines, which induce apoptosis of alveolar macrophages [59]. Much like CD4+ T-cells, macrophages play a fundamental role in the immune response to *Pneumocystis* via proinflammatory cytokines and chemokines; however, while these are effective at organism elimination, the response can come at a cost of pulmonary injury [55].

### 2.4. Neutrophils

Neutrophils are predominantly involved in inflammation rather than clearance of *Pneumocystis* organisms. Elevated neutrophil counts are correlated to a decrease in pulmonary function in HIV+ patients with PCP [60,61]. Mouse studies analyzing neutrophil function in knockouts of the NADPH oxidase component gp91(phox), a double knockout of gp91(phox) and inducible nitric oxide synthase, and a knockout of CXCR2, as well as antibody-induced neutrophil depletion have been examined [62]. In each of these models, no difference in *Pneumocystis* organism burdens, respiratory rates, arterial oxygen partial pressures, and intra-alveolar albumin concentrations were observed. Overall, neutrophils appear to be a marker of lung damage, but are not involved in causing lung damage in the mouse model.

### 2.5. B-Cells

B-cells play an essential role in the immune response to PCP. Using mixed bone marrow chimeric mice that lack expression of MHC class II on B-cells, rendering them unable to present antigen to CD4+ T-cells, an increase in susceptibility to infection is observed [63]. The increased susceptibility is likely due to diminished antibody production against *Pneumocystis*, and a lack of B-cell antigen presentation [63]. Additionally, patients with hyper-IgM syndrome have increased susceptibility to PCP [64,65]. This phenomenon has been observed in the clinic, whereby a 5-month-old infant tested positive for PCP with no known previous medical conditions, and it was later determined that the infant suffered from hyper-IgM syndrome [66]. In addition, another study examining a cohort of patients with a CD40 deficiency that resulted in hyper-IgM syndrome had a PCP incidence rate of 27% [64].

### 2.6. Natural Killer Cells

The role of natural killer (NK) cells in *Pneumocystis* clearance is poorly understood. Low numbers of NK cells or impaired NK cell function in HIV+ individuals have been suggested as a correlate of increased incidence of PCP [67,68,69,70]. Depletion of CD4+ T cells in mice leads to a decrease in NK cells in lung tissue, and the NK cells remaining have decreased upregulation of NKp46 and production of IFN-γ [71]. An increase in fungal killing occurs when NK cells are combined with CD4+ T cells compared to each cell type alone [71]. Furthermore, adoptive transfer of memory CD4+ T cells is required for NK cell upregulation of activation maker NK group 2D, and production of IFN-γ, granzyme B, and perforin during *Pneumocystis* infection in mice [71].

## 3. Drug Treatments

### 3.1. Current Drugs in Use

**Figure 1:**
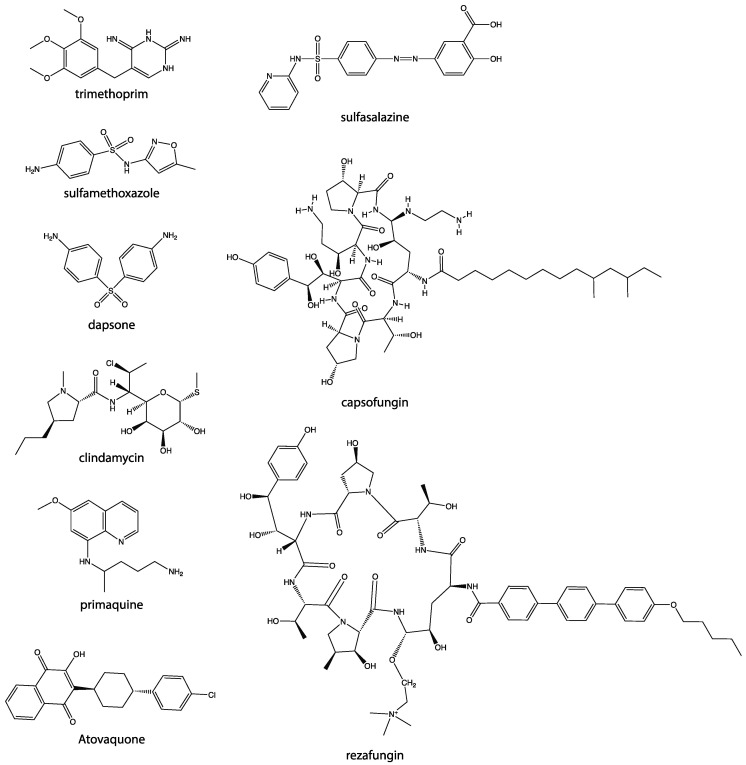
Chemical structures of drugs currently in use or in clinical trials for prevention and treatment of *Pneumocystis* infection.

A variety of drugs are currently available for the prevention and treatment of *Pneumocystis* infection (Figure 1). Trimethoprim-sulfamethoxazole (TMP-SMX) is the primary option for the prevention of PCP, and for treatment of mild to severe cases of PCP [72,73,74]. However, treatment side effects and increasing drug resistance are a major concern [75]. Adverse reactions are relatively frequent and can result in a number of conditions, including rashes, fever, gastrointestinal complications, cytopenia, marrow suppression, hyperkalemia, hepatoxicity, interstitial nephritis, aseptic meningitis, anaphylaxis, renal insufficiency, and pancreatitis [76]. Trimethoprim is a dihydrofolate reductase inhibitor, and sulfamethoxazole is a dihydropteroate synthetase inhibitor, and these drugs have a synergistic effect [77,78]. Trimethoprim binds to dihydrofolate reductase and inhibits the reduction of dihydrofolic acid to tetrahydrofolic acid, while sulfamethoxazole inhibits dihydropteroate synthase [79]. Both drugs interfere with the thymidine synthase pathway, which ultimately inhibits DNA synthesis of the organism [79]. Mutations to the dihydrofolate reductase (DHFR) and dihydropteroate synthase (DHPS) have been reported in *P. jirovecii*, possibly leading to treatment failure [80]. For patients with a sulfa-allergy, trimethoprim-dapsone can be used; however, in patients who have had serious reactions to TMP-SMX, dapsone is avoided as it can lead to fatal idiosyncratic dapsone-hypersensitivity syndrome [76]. Clindamycin-primaquine has been suggested for patients who have severe disease who are unable to take TMP-SMX [81]. Atovaquone can also be used in the treatment of mild cases of PCP, but is less effective than TMP-SMX in clinical trials [82]. Furthermore, drug resistance to atovaquone has begun to occur, as mutations in the cytochrome b gene have led to prophylactic failure of atovaquone [83].

While most drug treatments focus on targeting the organism, the use of sulfasalazine takes a different approach. Sulfasalazine is a disease-modifying antirheumatic drug used therapeutically in patients with rheumatoid arthritis [84]. While the mechanism of action of sulfasalazine has not been fully elucidated, its immunomodulatory and anti-inflammatory effects have been effective in treating PCP in mice by attenuating pulmonary inflammation and increasing fungal clearance [56]. The fungal clearance is driven by the ability of sulfasalazine to alter the T-cell response and drive macrophages to an M2 phenotype, leading to killing of *Pneumocystis* organisms [56,57,85]. In addition to these drugs, the anti-inflammatory properties of corticosteroids have improved the clinical outcomes of patients with moderate to severe PCP by limiting pulmonary damage [86]. However, such steroids must be used with care as non-HIV patients on corticosteroid regiments have an increased risk of developing PCP [87]. This observation has been supported in cancer patients receiving corticosteroids [88].

### 3.2. Drugs in Development

In addition to these current treatments already in use, newer drugs have been examined in preclinical mouse models and are now in clinical trials. Caspofungin, an echinocandin and competitive inhibitor of for 1,3-β-D-glucan synthesis, which is a major component of the fungal cell wall, is effective at reducing the cystic form of *Pneumocystis* and enhancing survival of mice [89]. However, the drug does not eliminate the trophic reservoir, which leads to cyst reformation following treatment withdrawal. Numerous reports of caspofungin alone and in combination with TMP-SMX have been reported, with most indicating efficacy in treatment [90]. Based on these data, two clinical trials are ongoing to determine the efficacy of caspofungin in non-HIV patients with PCP (NCT02603575, NCT03978559). Another echinocandin, rezafungin [91], has shown prophylactic efficacy against *Pneumocystis* in an immunosuppressed mouse model by a reduction in nuclei and asci counts [92]. In addition, rezafungin was shown to limit *Pneumocystis* reactivation following cessation of therapy in the same mouse model [93]. A current phase III clinical trial is ongoing to determine the efficacy of rezafungin in allogenic blood and marrow transplant patients (NCT04368559).

Due to the immunocompromised state of individuals who develop PCP, utilization of passive immunization with antibodies is a viable potential therapeutic option. The first attempt at determining the effectiveness of antibody treatment demonstrated that the mAb M5E12 conferred partial protection against PCP in drug-induced immunosuppressive mouse and ferret animal models [94]. Mice born to *Pneumocystis*-immunized dams have higher antibody titers compared to mice born from naïve dams, and this increase in antibody levels is correlated with enhanced *Pneumocystis* clearance in challenged mice [95]. The use of hyperimmune serum in both mouse and rat models was shown to be protective in two independent studies reinforcing the effectiveness of antibodies against PCP [96,97]. A promising avenue of treatment is the use of combination therapy to treat PCP. It was recently shown that a cocktail of antibody and sulfasalazine leads to a dramatic improvement in the severity of PCP in a mouse model compared to single therapy methods; however, the mechanism by which this occurs needs further investigation [98].

## 4. Vaccine Development against PCP

### 4.1. Vaccination with Whole Organisms

Some of the first attempts at developing vaccines for *Pneumocystis* utilized inactivated whole organisms [53,99,100]. Since *Pneumocystis* is an opportunistic pathogen, the animal models used must undergo immunosuppression following vaccination for efficient infection. *Pneumocystis* has a very specific host range, demonstrated by a study immunizing mice with either mouse- or ferret-derived *Pneumocystis*. Mice immunized with mouse-derived *Pneumocystis* demonstrated a robust protective effect upon intratracheal infection with *Pneumocystis*, yet mice immunized with ferret-derived *Pneumocystis* were not protected [101]. Another study utilizing non-viable organisms along with cholera toxin fraction B administered intranasally was found to be protective and generated robust IgA and IgG antibody titers [99]. These results are promising for utilizing whole organisms in vaccinations; however, a major hurdle in this approach is the inability to grow *Pneumocystis* in vitro, limiting this approach from being effective on a large scale. Overall, while immunization with whole *Pneumocystis* organisms has been shown to be quite effective in animal models [53,99,100], the growth limitation has led to the exploration of subunit-based vaccines [102].

### 4.2. Subunit Vaccines

Below, we summarize the protein candidates that have been examined as vaccine antigens in *Pneumocystis* subunit vaccines (Table 1) [99,100,101,102,103,104,105,106,107,108,109,110,111,112,113,114,115,116,117,118,119,120,121,122,123,124,125,126,127,128].

**gpA (Msg):** The major surface glycoprotein (Msg or glycoprotein A (gpA)) is an important B and T cell target following immunization with whole organisms [103,104]. However, gpA has enormous amino acid diversity, which is likely a result of evolutionary immune evasion [104,105]. Mice vaccinated with gpA demonstrate a robust specific antibody response, but are not protected from infection [102]. An additional study in the rat model showed that animals vaccinated with gpA saw results with variable levels of protection [106]. It was further demonstrated that immunization with gpA leads to cross-reactive antibody responses to gpA variants, but not cross-reactive T-cells, suggesting variation in gpA by *Pneumocystis* is used for T-cell escape [107]. Humans generate antibodies to gpA, and this protein is predominantly used in *Pneumocystis* serological studies, as antibodies to gpA increase following PCP [108,109,110,111]. Due to the genetic variation in gpA, the protein is not actively being pursued as a vaccine candidate.

**p55:** A 55 kDa protein antigen, termed p55, resides inside the *Pneumocystis* cell wall, and both natural and recombinant p55 proteins can stimulate a protective immune response [112,113]. p55 protein from rat-derived *Pneumocystis* antigen has >80% sequence homology with nonhuman primate (NHP) and human variants of the protein [113,114]. A p55-based multi-epitope antigen incorporating 12 B-cell epitopes elicits both cell and humoral immune responses in rats, and vaccination with this antigen followed by dexamethasone immunosuppression to induce *Pneumocystis* infection results in reduced fungal burden [114]. In another study utilizing methylprednisone for rat immunosuppression, immunization with p55 led to a decrease in organism burden, improved histology scores, and reduced inflammation, yet only partial protection against PCP was observed [112]. In a T-cell independent approach, an adenovirus 5 vector encoding murine CD40L was used to modify dendritic cells to express CD40L, and these cells were then pulsed with *Pneumocystis* organisms ex vivo. Mice immunosuppressed with an anti-CD4 monoclonal antibody were then administered the modified dendritic cells. This immunization lowered the organism burden in challenged mice, and antibodies generated as a result of vaccination induced opsonic killing via a macrophage-mediated mechanism [115]. The antibody response generated by this vaccination led to p55-specific IgG [115].

**A12 (Pca1):** The A12 antigen was discovered using a cross-reactive mouse monoclonal antibody, 4F11, that is cross-reactive against multiple *Pneumocystis* spp. [116]. The 142-AA A12 polypeptide elicits a protective response as a vaccine antigen, and facilitates a decrease in *Pneumocystis* organism burden and lung inflammation in a mouse model [117]. Following identification of the full-length protein containing the A12 sequence, the entire molecule was renamed *Pneumocystis* cross-reactive antigen 1 (Pca1). Active immunization with the N-terminal half of Pca1 is protective in a mouse model. Interestingly, although sharing limited sequence identity with homologs with other *Pneumocystis* spp., Pca1 is able to induce antibodies that are able to cross react with *P. jirovecii* [118].

**Kexin:** Kexin is a serine protease that shares sequence homology with kexin proteins from other fungal pathogens, and was initially identified by probing a cDNA library with monoclonal antibodies [119]. Intranasally administered anti-kexin monoclonal antibodies are protective in an SCID mouse model of PCP [120]. Several studies have investigated the utility of kexin as a candidate vaccine. DNA vaccination of anti-CD4 antibody depleted mice with full length kexin and mouse CD40L (to induce a CD4-independent response) results in anti-pneumocystis antibody titers in CD4+ T cell-depleted mice [115]. This immunization lowers the organism burden in challenged mice, and antibodies generated as a result of vaccination induce opsonic killing via a macrophage-mediated mechanism [115]. A subunit recombinant protein vaccine consisting of a 90 amino acid fragment of Kexin (KEX1) has also been examined as a potential candidate. In a NHP model of HIV and PCP, rhesus macaques vaccinated with KEX1 followed by simian-human immunodeficiency virus (SHIV) infection and *Pneumocystis* challenge by exposure to *Pneumocystis*-colonized animals are protected from developing PCP up to 36 weeks after immunosuppression [121]. In a separate model using methylprednisone-immunosuppressed rhesus macaques as a model for drug-induced immunosuppression, KEX1 vaccination prior to immunosuppression produces a robust and enduring antibody response that continues throughout immunosuppression, and boosting with KEX1 during the immunosuppression phase induces recall of memory responses against KEX1 [122].

KEX1 has also been examined as a serological marker for the risk of developing PCP. Most adults have been exposed to *P. jirovecii*, based on studies demonstrating children are seropositive by the age of four [123,124,125]. In a retrospective study of AIDS patients comparing those who developed PCP and those who developed a non-PCP AIDS-defining illness, those who developed PCP were more likely to have low KEX1 IgG prior to PCP diagnosis [126]. It was concluded that antibody levels to KEX1 are predictive of HIV patients developing PCP, as those with elevated antibody levels specific to KEX1 had a reduced risk of infection [126]. This observation was further confirmed in a nonhuman primate model of HIV and *Pneumocystis* coinfection, whereby higher baseline plasma levels of KEX1-IgG were correlated with decreased incidence of *Pneumocystis* colonization [127].

**SPD1:** Utilizing a chemical labeling technique to identify surface proteins as potential vaccine candidates led to the antigen SPD1 being identified in *P. murina* [128]. SPD1 has high sequence identity with homologs from *P. jirovecii*. Mice immunized with SPD1 develop IgG antibody responses and an increase in memory B-cells following *Pneumocystis* challenge. In addition, serum from rhesus macaques exposed to *Pneumocystis* reacts to SPD1. Vaccination of mice with N-terminal and C-terminal fragments of SPD1 shows no effect in CD4+ T cell-depleted mice at 1-week post vaccination. However, a decrease in organism burden is observed at 4 weeks for the C-terminal fragment and at 6 weeks for both fragments. These data suggest SPD1 is expressed on the surface of *Pneumocystis* organisms later during the infection time course, and/or there is a lag time in the development of functional antibodies to SPD1.

## 5. Conclusions

PCP remains a significant public health concern. At the advent of the HIV epidemic, the incidence of PCP increased rapidly, and as treatments to control HIV infection gained widespread use and high efficacy, the incidence of HIV patients diagnosed with PCP decreased. However, PCP remains an AIDS-defining illness with a consistently high mortality rate [129]. In addition, cases of non-HIV-associated PCP are now more prominent due to increased usage of immunosuppressive and immunomodulating drugs [13,130,131]. While drugs to prevent the development of PCP, primarily TMP-SMX, have been generally successful, major concerns remain in those patients who are diagnosed with PCP. Clinical trials for additional antifungal drugs are currently ongoing, and antibodies have shown promise in preclinical models, especially when combined with other drugs. An ideal course of action would be development of a vaccine for prevention of PCP, which could be selectively administered to those at high risk of HIV infection, and those individuals who have planned procedures or drug regimens that will lead to immunosuppressive states. Several vaccine candidates have shown promise in preclinical models, primarily in mouse models, and the KEX1 candidate has proven effective in immunosuppressed NHPs in both HIV-associated and drug-induced immunosuppression. Since *Pneumocystis* species have restricted host specificity, those vaccine candidates that have high conservation in *P. jirovecii* would presumably have the highest chance of success, yet it is unclear if such vaccine candidates are able to protect against *P. jirovecii*. However, the inability to grow *Pneumocystis* in vitro and the lack of a genetically tractable system limits the ability to examine if vaccine candidates that have shown efficacy in preclinical models have the same function in *P. jirovecii*. Recent advancements in single-cell sequencing and immune profiling may assist these efforts by identifying genes expressed at each stage of the *Pneumocystis* life cycle, and identifying additional monoclonal antibody candidates and T cell epitopes for therapy design and vaccine development. Overall, vaccine and therapeutic development has made promising advances in recent years, although significant hurdles remain to advance preclinical studies to human clinical trials.

## Figures and Tables

**Table 1:** Summary of preclinical vaccine studies for prevention of *Pneumocystis* infection.

Antigen	Animal Model	Dose Route Adjuvant	Immunosuppression Method	Protective?	Efficacy Readout (Result)	Ref
Inactivated whole organism	Murine	10^6^ trophs IN Cholera toxin B	CD4 T-cell depletion via mAb	Partial	i. Stained lung smears (no organisms present) ii. PCR (positive for mitochondrial rRNA)	[99]
	Murine	10^7^ organisms IT None	CD4 T-cell depletion via mAb	Partial	i. Stained lung smears (decreased organism burden)	[100]
	Murine	10^7^ nuclei IT None	CD4 T-cell depletion via mAb	Yes	i. Stained lung smears (no organisms present) ii. PCR (negative for glycoprotein A and mitochondrial rRNA)	[53]
	Murine	10^6^ cysts IT None	CD4 T-cell depletion via mAb	Yes	i. Stained lung smears (no organisms present)	[101]
gpa (Msg)	Murine	10-10 µg IT Quil A	CD4 T-cell depletion via mAb	No	i. Stained lung smears (organisms present)	[102]
	Rat	1-100 µg SubQ Titermax	Methylprednisolone	Partial	ii. Stained lung smears and sections (Decrease in organism burden)	[109]
p55	Rat	100 µg SubQ Titermax	Methylprednisolone	Partial	i. Stained lung smears and (Decrease in organism burden)	[112]
	Rat	100 µg IM None	Dexamethasone	Partial	i. Stained lung sections (Decrease in organism burden) ii. PCR (Positive for p55)	[114]
	Murine	10^4^ Pc-pulsed DCs IV None	CD4 T-cell depletion via mAb	Partial	i. Stained lung sections (no organisms present) ii. PCR (Positive for mitochondrial rRNA)	[115]
A12 (Pca1)	Murine	25 µg SubQ Titermax	CD4 T-cell depletion via mAb	Partial	i. PCR (10/14 mice negative for single-copy kex1 gene) ii. PCR (Positive for gpA gene)	[117]
	Murine	100 µg SubQ Titermax	CD4 T-cell depletion via mAb	Partial	i. PCR (Positive for single-copy kex1 gene) ii. PCR (negative for multicopy gpA gene)	[118]
SPD1	Murine	5 µg SubQ MF-59	CD4 T-cell depletion via mAb	Partial	i. PCR (reduction in mitochondrial rRNA copy number)	[128]
Kexin	Rhesus macaques	50-100 µg IM Alum	Simian immunodeficiency virus	Partial	Criteria for PCP (1/6 animals developed PCP): i. BAL smears (detection of organism in BAL fluid) ii. PCR (PCR of organism DNA in BAL) iii. Immunohistochemistry (organism detection in lungs)	[121]
IN—intranasal, IT—intratracheal, SubQ—subcutaneous, IM—intramuscular						
